# Impact of VSD therapy on surgical outcomes, inflammatory markers, and functional rehabilitation in patients with secondary bone infection following tibial fracture surgery

**DOI:** 10.3389/fcimb.2024.1508424

**Published:** 2025-01-17

**Authors:** Sheng Chang, Yu Wang, Zengshan Zhang

**Affiliations:** ^1^ Department of Spine Surgery, Heze Municipal Hospital, Heze, China; ^2^ Department of Anesthesiology, Women’s and Children's Hospital Affiliated to Qingdao University, Qingdao, China; ^3^ Department of Orthopedics, Affiliated Hospital of Shandong Second Medical University, Weifang, China

**Keywords:** VSD, bone infection secondary to tibial fracture, distribution of pathogenic bacteria, inflammatory factors, bone infection

## Abstract

**Objective:**

The aim of this study was to investigate the effect of vacuum sealing drainage (VSD) treatment on surgical indicators, inflammatory factors, and functional recovery in patients with chronic osteomyelitis secondary to open tibial fractures.

**Methods:**

In total, 87 patients with secondary bone infection after internal fixation of tibial fracture treated in the Affiliated Hospital of Shandong Second Medical University from December 2020 to June 2022 were selected, all of whom were tibial shaft fractures. Of these, 55 cases of primary open fracture were sutured in the first stage; 32 cases underwent internal fixation after primary debridement at the time of trauma. The patients were treated with surgical debridement, removal of internal fixation, and fixation with an external fixation frame. After debridement, those with local wounds that could not be completely closed and were complicated with exposed bone were randomly selected for either VSD covering treatment (study group, n=46) or bone cement covering treatment (control group, n=41. The distribution of pathogenic bacteria, surgical indicators, inflammatory factors [tumor necrosis factor⁃α(TNF⁃α), interleukin⁃6 (IL⁃6), and C⁃reactive protein (CRP) levels], functional recovery [knee, ankle, and limb function recovery], and complications were summarized.

**Results:**

There were 87 pathogenic bacteria strains in 87 patients, including 43 Gram⁃positive bacteria strains (49.42%), 32 Gram⁃negative bacteria strains (36.78%), and 12 fungi strains (13.80%). The number of dressing changes in the study group was less than that in the control group. The infection control time, wound sterility time, hospitalization time, and skin flap transfer operation time in the study group were shorter than those in the control group and the difference was statistically significant (P<0.05). After treatment, the levels of TNF⁃α, IL⁃6, and CRP in the two groups decreased, among which the change in the study group was the most significant and the difference between the two groups was statistically significant (P<0.05). After treatment, the Hospital for Special Surgery Knee Score and Baird–Jackson score of the two groups increased, among which the change in the study group was the most significant and the difference between the two groups was statistically significant (P<0.05). The excellent and good rate of the study group (95.65%) was higher than the excellent and good rate of the control group (80.49%) and the difference was statistically significant (P<0.05).

**Conclusion:**

When a wound cannot be closed, VSD treatment of patients with secondary bone infection after internal fixation of tibial fracture can improve the level of surgical indicators and inflammatory factor levels in patients, and promote the recovery of patients’ limb function, and is thus worthy of clinical promotion and application.

## 1 Introduction

Tibial fractures, especially in the middle and lower segments, are common orthopedic issues with treatment and recovery challenges. Limited soft tissue cover makes the tibia prone to fracture upon trauma, potentially causing open fractures. Treatment is prolonged, complex, and risky due to the tibia’s proximity to the skin and poor blood supply (Li and Wang, 2018). The management of tibial fractures typically involves a two-stage approach. In the initial phase, the risk of infection is controlled through appropriate wound care and antibiotic therapy. Once the infection risk has been mitigated, internal fixation surgery is performed to stabilize the fracture and facilitate healing. However, despite meticulous care and surgical intervention, local infections can still arise due to systemic or local factors post-surgery, leading to refractory bone infections (Wang et al., 2017).

Bone infections, primarily caused by bacteria such as *Staphylococcus aureus*, *Escherichia coli*, and *Pseudomonas aeruginosa*, infect bone tissues such as bone marrow and periosteum, leading to inflammation and symptoms including wound pus, redness, swelling, pain, fever, and bone necrosis. In tibial fracture patients, these infections can have severe consequences, impeding wound healing and fracture union, and potentially causing physical dysfunction and disability. Prompt and effective clinical intervention is crucial to prevent complications such as osteomyelitis or septic arthritis, which can profoundly impact patients’ quality of life, physical health, and mental well-being. The primary treatment involves surgical intervention, including debridement, irrigation, and antibiotic therapy. However, some patients may not fully recover post-surgery and face a higher risk of complications or recurrence. Recently, vacuum sealing drainage (VSD) has shown promise in treating complex wounds, including those associated with bone infections, by creating a sealed environment that removes exudate, reduces edema, and stimulates granulation tissue formation (Jiang et al., 2020; Deng et al., 2023).

In this comprehensive study, we aim to delve into the effects of VSD treatment on surgical indicators, inflammatory factors, and functional recovery in patients who have developed postoperative bone infections secondary to tibial fracture. By comparing the outcomes of patients treated with VSD to those treated with traditional surgical methods, we hope to gain insights into the potential benefits of VSD in managing this challenging condition. The findings of this study will contribute to the evidence base for the use of VSD in the treatment of postoperative bone infections and may ultimately lead to improved patient outcomes and quality of life.

## 2 Information and methodology

### 2.1 General information

In total, 87 patients with bone infection secondary to internal fixation of tibia fracture admitted to the Affiliated Hospital of Shandong Second Medical University from December 2015 to June 2024 were selected, all of them were tibial stem fracture; among them, 55 were cases of original open fracture, all of which were sutured in one stage, and 32 cases were cleared in one stage at the time of traumatic injuries and were then subjected to internal fixation. The patients were treated with surgical debridement, removal of internal fixation, and fixation with an external fixation frame, and, after debridement, there were cases in which the wound could not be closed completely and the bone was exposed. These patients were randomly selected to be treated with VSD cover (study group, n=46) or cement cover (control group, n=41), and their indexes were summarized and analyzed. In the study group, there were 25 men and 21 women, with an average age of 35.28 ± 3.26 years; in the control group, there were 23 men and 18 women, with an average age of 34.37 ± 4.49 years. There was no statistically significant difference between the general information of the two groups (P>0.05).

Inclusion criteria: ① in an imaging examination, meet the diagnostic criteria of bone infection (Chastain and Davis, 2019) of local sinus tract formation; ② bone infection after debridement to form a trauma, skin defects, and bone exposure; ③ after one debridement treatment, the trauma surface is clean and the local flap transfer is healing (i.e., infection control); ④ in the hospital and complete clinical data with no defects or loss; ⑤ the patients and their families gave informed consent to the study. The study was approved by the ethics committee of the hospital and the patients and their families were informed and agreed to the study.

Exclusion criteria: (1) people with intact skin and soft tissues; (2) people who had one debridement surgery and the infection was not controlled; (3) people who had necrosis of the flap or the as was not healing for more than 3 weeks after the flap transfer (the infection was not controlled); (4) people who dropped out of the study; (5) people who had systemic acute toxicity symptoms, such as fever, and could not undergo the operation, or people who have a weak body and could not tolerate the operation; (6) people who could not cooperate with this study because of mental illness or related family history.

### 2.2 Methods

#### 2.2.1 Pre-operative

The patients were admitted to the hospital for a routine pre-operative examination, and at the same time, blood sedimentation, C-reactive protein (CRP), tumor necrosis factor⁃α (TNF⁃α), and interleukin⁃6 (IL-6) laboratory tests were performed, bacterial cultures and drug sensitivity tests were conducted, and sensitive antibiotics were administered according to the results.

VSD operation process: First, the dressing matching the size of the wound was cut according to the size and shape of the wound. A small hole was cut in the central position of the dressing, and the side hole end of the drainage tube was embedded in it. Then, the foam dressing with a drainage catheter was filled in the wound of the patient. The strength of the filling was measured to avoid secondary trauma to the wound of the patient and reduce the pain and discomfort of the patient during the operation. At the same time, the foam dressing was filled completely to minimize the residual cavity and dead angle in the wound. Second, whether to place an irrigation catheter was determined according to the cleanliness of the wound. If there were more viscous secretions and necrotic tissue in the wound, an irrigation catheter was placed. The catheter was placed between the wound and the dressing, and it established a good circulation state. In addition, the patient’s wound was closed with a transparent 3M film so that the patient’s wound formed a closed system isolated from the external environment. Finally, the successful operation of the VSD device was confirmed when the negative pressure suction device was connected to the other end of the drainage catheter and the negative pressure was turned on. If the foam dressing and the sealing film were slightly deflated, the VSD device was successfully operated.

#### 2.2.2 Study group

The bone surface was covered with petroleum jelly gauze, and according to the patient’s trauma, the VSD was pre-trimmed to cover and fill the bone defect area and all the skin of the surgical incision. After cleaning the skin around the trauma, the trauma was closed with biofilm, and the negative pressure drainage was carried out for 1 week, and then the dressing was taken out, and the trauma infection control was observed.

#### 2.2.3 Control group

The wound is covered with bone cement and bandaged with a sterile dressing. After the oozing around the bone cement was reduced and basically dry, the dressing was removed and the wound was observed.

#### 2.2.4 End of the study node

Local flap transfer was carried out after infection control through comprehensive judgment utilizing wound observation, secretion cultures, laboratory indexes, and clinical experience, and the flap was healed without necrosis within 3 weeks.

### 2.3 Observation indicators

#### 2.3.1 Distribution of pathogen composition

Before treatment, 87 patients underwent bacterial culture and drug sensitivity tests. Each patient had two specimens collected within 3 hours, and was routinely cultured. Pure colonies were selected and identified by a bacterial identifier (VITEK 2 Compact, BioMérieux, France), and the common bacterial groups obtained from the two specimens were localized to the pathogens, and the culture of the specimens was carried out in accordance with the National Clinical Laboratory Practice Guidelines (Tudan, 2023). The isolation and identification of pathogenic bacteria and drug sensitivity experiments were carried out using the French NEW and ATB analyzers, and the quality control strains were *S. aureus* (ATCC29213), *E. coli* (ATCC25922) and *P. aeruginosa* (ATCC27853).

#### 2.3.2 Surgical indicators

The surgical indicators included time to control infection, length of wound asepsis, number of dressing changes, time to perform flap transfer, and length of hospitalization.

#### 2.3.3 Inflammatory factor

Before the patients were admitted to the hospital for surgical treatment and 1 week after the operation (VSD time was removed), 5 mL of morning fasting venous blood was collected from all the participants, and the serum was separated by centrifugation at 3,000 r/min, with a centrifugation radius of 9 cm, and taken out after 10 min for cryopreservation. Tumor necrosis factor⁃α, interleukin⁃6, and C-reactive protein levels were measured using enzyme-linked immunoluminescence.

#### 2.3.4 Body function

The Hospital for Special Surgery Knee Score (HSS) and the Baird–Jackson Functional Score were used to score the patients’ knee and ankle joints, and the higher the score, the better the recovery of joint function (Mora-Zúñiga et al., 2021). The patients were scored according to Paley’s criteria, which included four grades: excellent, good, acceptable, and poor, and the excellent rate = [(excellent+good)/total number of cases]×100%. Paley’s criteria for functional recovery evaluation are as follows: ① obvious claudication; ② ankle stiffness with a horseshoe shape; ③ soft tissue dystrophy; ④ limb pain; ⑤ limb motor dysfunction. Level 1: none of the symptoms mentioned above, good range of motion of limbs; Level 2: ① to ④ 1-2 symptoms, good physical activity; Grade 3: ① to ④ symptoms of 3-4, good physical activity; Level 4: the fifth symptom is satisfied.

### 2.4 Statistical methods

SPSS 22.0 statistical software was used for statistical analysis. The measurement data were described by mean ± standard deviation (x ± s) and analyzed by t-test. Count data were expressed by n (%) and χ2 test was performed. P<0.05 was regarded as the difference of statistical significance.

## 3 Results

### 3.1 Distribution of pathogen composition

A total of 87 strains of pathogenic bacteria were found in 87 patients, including 43 (49.42%) Gram-positive, 32 (36.78%) Gram-negative, and 12 (13.80%) fungal strains, as shown in **Table 1**.

**Table 1:** Comparison of the composition and distribution of pathogens between the two groups.

Pathogen		Number of strains	Composition ratio (%)
Gram-positive bacteria		43	49.42
	*Staphylococcus aureus*	26	29.88
	Coagulase-negative *staphylococcus*	12	13.79
	*Enterococcus*	1	1.15
	Other	4	4.60
Gram-negative bacteria		32	36.78
	*Escherichia coli*	19	21.84
	*Pseudomonas aeruginosa*	6	6.90
	*Acinetobacter baumannii* (immobile bacteria)	3	3.45
	*Enterobacter cloacae*	1	1.15
	Other	3	3.45
Fungi		12	13.80
	*Pseudomonas albicans*	8	9.19
	*Pseudomonas tropicalis* (a species of yeast)	2	2.30
	Pseudohyphae	1	1.15
	Other	1	1.15
Total		87	100

### 3.2 Surgical indicators

The number of dressing changes in the study group was lower than that in the control group, and the time taken for infection control, wound asepsis, hospitalization, and flap transfer surgery was shorter than those in the control group, with statistically significant differences (P<0.05), as shown in **Table 2**.

**Table 2:** Comparison of surgical-related indicators between the two groups (x ± s).

Groups	n	Duration of infection control (d)	Duration of wound sterility (d)	Time to perform flap transfer (d)	Length of hospitalization (d)	Number of medication changes (times)
Study group	46	11.32 ± 3.62	25.82 ± 6.17	8.14 ± 3.21	34.13 ± 2.64	2.35 ± 0.71
Control group	41	20.38 ± 5.84	40.55 ± 7.63	16.17 ± 4.15	43.97 ± 4.45	8.17 ± 1.26
t		8.798	9.945	10.153	12.703	26.957
P		<0.001	<0.001	<0.001	<0.001	<0.001

### 3.3 Inflammatory factor

The levels of TNF⁃α, IL⁃6, and CRP decreased in both groups after treatment, with the most significant changes in the study group (P<0.05).

In the study group, the most significant change was found, and the difference was statistically significant (P<0.05), as shown in **Table 3**.

**Table 3:** Comparison of inflammatory indicators between the two groups before and after treatment (x ± s).

Groups	n	TNF⁃α (pg/mL)		IL⁃6 (pg/mL)		CRP (mg/dl)	
		Before	After	Before	After	Before	After
Study group	46	278.76 ± 27.82	93.19 ± 15.43	361.54 ± 9.88	128.31 ± 13.09	1.89 ± 0.15	0.75 ± 0.17
Control group	41	279.13 ± 28.74	165.32 ± 16.22	362.12 ± 8.06	175.52 ± 15.38	1.87 ± 0.13	1.16 ± 0.14
t		0.059	21.245	0.298	15.465	0.661	12.19
P		0.953	<0.001	0.767	<0.001	0.511	<0.001

P<0.05 compared with the same group before treatment.

### 3.4 Body function

The HSS and Baird–Jackson score increased in both groups after treatment, with the most significant change in the study group, and the difference was statistically significant (P<0.05). The good rate of the study group was higher than that of the control group (95.65%), and the difference was statistically significant (P<0.05), as shown in **Table 4**.

**Table 4:** Comparison of limb function between the two groups before and after treatment [(x ± s), n(%)].

Groups	n	HSS		Baird–Jackson		Excellent Rate
		Before	After	Before	After	
Study group	46	36.14 ± 10.73	60.41 ± 8.19	63.57 ± 7.63	91.49 ± 4.31	44 (95.65)
Control group	41	36.87 ± 10.64	49.36 ± 10.63	63.04 ± 7.17	83.31 ± 6.62	33 (80.49)
t		0.318	5.463	0.333	6.901	4.900
P		0.751	<0.001	0.74	<0.001	0.027

P<0.05 compared with the same group before treatment.

## 4 Discussion

The tibia, a crucial long bone, is prone to open fractures from an external force, leading to clinical treatment challenges and a high risk of bone infections. Open fractures often result in poor soft tissue conditions, joint stiffness, and ligament contraction, affecting recovery (Yisireyili et al., 2017). Postoperative bone infections are a critical issue in orthopedic clinics due to long treatment times, high disability rates, and poor outcomes. Antibiotic bone cement can locally prevent and treat infections, but its effectiveness is limited in tibial fracture patients with secondary bone infections due to soft tissue defects and biofilm-protected bacteria (Christersson et al., 2018; Ma et al., 2022). This study found similar pathogen distributions as previous research, with Gram-positive bacteria (49.42%), Gram-negative bacteria (36.78%), and fungi (13.80%). Advances like the VSD technique have improved bone infection cure rates by creating a sealed negative pressure environment that promotes wound healing, reduces secondary infections, and enhances microcirculation (Krappinger et al., 2015). The study showed significant improvements in inflammatory levels and limb function in the VSD group, consistent with prior research (Ma et al., 2018). VSD reduced surgery and treatment times, enhanced recovery, and minimized drug side effects and patient pain. However, limitations include a small sample size (87 patients), potential bias from random group assignment and a single-center location, short follow-up time, and diverse pathogen distribution. Future research should expand sample sizes, include multi-center studies, and conduct long-term follow-ups to fully evaluate VSD’s efficacy and safety, especially in patients with specific pathogens (Huang et al., 2021).

To reduce potential bias, future studies should further optimize randomization and blinding design. For example, a double-blind or triple-blind design could be used to ensure objectivity and accuracy of the results. Future studies should extend the follow-up period to fully evaluate the long-term efficacy of VSD techniques. At the same time, we should pay attention to the quality of life of patients, the incidence of complications, and other indicators to understand the clinical value of VSD technology more comprehensively. Future studies could focus on the efficacy and safety of VSD in patients infected with specific pathogens, such as drug-resistant bacteria. More effective treatment options could be developed for these patients through subgroup analysis or specialized studies. Future studies could explore the combination of VSD technology with other treatment methods (such as antibiotic bone cement, and membrane induction technology). By optimizing the treatment plan, the efficacy and safety of patients with secondary bone infection after tibial fracture surgery can be further improved. In conclusion, if the wound cannot be closed, VSD can improve the surgical index and inflammatory factor level of the patients, and promote the recovery of limb function after internal fixation of tibial fracture, and is thus worth popularizing and applying in the clinic.

## Data Availability

The original contributions presented in the study are included in the article/supplementary material. Further inquiries can be directed to the corresponding author.
